# Therapeutic Plasmapheresis with Albumin Replacement in Alzheimer’s Disease and Chronic Progressive Multiple Sclerosis: A Review

**DOI:** 10.3390/ph13020028

**Published:** 2020-02-12

**Authors:** Rut Navarro-Martínez, Omar Cauli

**Affiliations:** 1 Haematology Department, Hospital General Universitario, 46014 Valencia, Spain; Rut.Navarro@uv.es; 2 Department of Nursing, University of Valencia, 46010 Valencia, Spain; 3 Frailty and Cognitive Impairment Group (FROG), University of Valencia, 46010 Valencia, Spain

**Keywords:** plasmapheresis, albumin, auto-immunity, dementia, magnetic resonance imaging, amyloid beta

## Abstract

Background: Reducing the burden of beta-amyloid accumulation and toxic autoimmunity-related proteins, one of the recognized pathophysiological markers of chronic and common neurological disorders such as Alzheimer’s disease (AD) and multiple sclerosis (MS), may be a valid alternative therapy to reduce their accumulation in the brain and thus reduce the progression of these disorders. The objective of this review was to evaluate the efficacy of plasmapheresis (PP) in AD and chronic progressive MS patients (in terms of improving clinical symptoms) and to analyze its safety and protocols. Methods: Articles related to this topic and published without time limitations in the Medline, and Cochrane databases were reviewed. Results: In AD patients, PP reduced amyloid beta (Aβ) levels in the brain, accompanied by a tendency towards cognitive stabilization, and improved language and verbal fluency. In regards to structural and functional brain changes, PP reduced brain volume and favored the stabilization, or absence, of the progression of perfusion. In chronic progressive form of MS patients, PP improved neurological deficits in 20–70% of patients with a chronic progressive form of MS, and restored interferon (IFN) responsiveness, which was not accompanied by any image change in brain plaques. Conclusions: Therapeutic plasmapheresis with albumin replacement is a promising strategy for reducing Aβ mediated toxicity and slowing the progression of the disorder. Some patients with chronic progressive forms of MS show improvement in neurological deficits. The features of AD and MS patients who benefit most from this approach need further research.

## 1. Introduction

Neurological disorders are increasingly being recognized as major causes of death and disability worldwide. A recent worldwide epidemiological study found the burden of neurological disorders, measured in terms of the absolute number of disability-adjusted life-years (DALYs), i.e., the sum of years of life lost and years lived with disability by age and sex, has increased for most neurological disorders in the last decade [1]. Among common neurological disorders, Alzheimer’s disease (AD) and multiple sclerosis (MS) are associated with high-morbidity levels and health costs [2,3]. To date, only symptomatic pharmacological treatments have been approved for the treatment of AD, including cholinesterase inhibitors and N-methyl-d-aspartate receptor antagonists, as the cornerstone of pharmacotherapy [4]. The amyloid beta (Aβ) peptide is the main protein component of the extracellular space found in senile plaque in the brain parenchyma, and is involved in memory dysfunction in AD [5,6]. Besides accumulation of Aβ peptide, other pathogenic hallmarks, such as neurofibrillary tangles, are responsible for the pathology of AD [7]. MS is a chronic-autoimmune disease of the central nervous system (CNS) which is most common in young female patients. Its pathophysiological hallmark is the destruction of the myelin sheath, with axonal degeneration and neuronal cell death. Furthermore, pharmacological treatment in MS is not curative, and is based on three goals: treatment of exacerbations, slowing the disease’s progression with disease-modifying therapies (DMTs), and symptomatic therapies [8]. Disease-modifying drugs have mostly failed as treatments for the clinical form of progressive MS [9] and there is a particular need for new strategies to treat patients with this form of MS. Management of progressive MS, therefore, merely aims to minimize the symptoms, prevent exacerbations, and if possible, improve function. Therapies aimed at preventing the accumulation of toxic substances in the blood (Aβ or autoantibodies), or in the brain, may have therapeutic uses in AD and MS patients. Reducing amyloid deposits or reducing the amount of plaque in the brain are currently being investigated for AD treatment [10,11,12]. Promising results pinpoint the reduction of the concentration of toxic substances associated with AD physiopathology, such as the Aβ peptide in the brain [13]. Therapeutic plasma exchange apheresis (PP) is an extracorporeal blood purification technique designed to remove substances with a large molecular weight. The utility of this procedure includes the removal of antibodies, alloantibodies, immune complexes, monoclonal proteins, toxins and cytokines, and it involves the replenishment of a specific plasma factor containing 5% albumin. PP has been successfully used in several immune-mediated neurological disorders, including Guillain–Barré syndrome, chronic inflammatory demyelinating polyneuropathy, and myasthenia gravis [14,15,16,17,18]. Less common neurological diseases in which plasmapheresis has afforded beneficial effects are paraneoplastic polyneuropathies, neuromyelitis optica (also known as Devic’s disease), motor neuron disease, polymyositis, and multifocal motor neuropathy [18]. PP can be a therapeutic strategy to remove or reduce the substances that are considered pathogenically responsible, e.g., Aβ peptides in AD from the blood, by changing their transportation through the blood-brain barrier, thereby limiting their accumulation in the brain. In the case of MS, eliminating pathogenic humoral factors from the blood [14], including suspected auto-antibodies directed against the myelin sheath, is needed in some patients with steroid refractory relapses [15], or in patients that develop neutralizing antibodies to interferon-beta (IFN-β), which are associated with reduced bioactivity and efficacy of IFN-β [19,20]. There is extensive literature related to the use of plasma exchange in relapsing and remitting multiple sclerosis, and its use as a temporary treatment of acute relapses in steroid-unresponsive MS patients has been recently reviewed [15,16]. For this reason, the aim of the review does not include the studies on the relapsing and remitting form of MS (the most common form of the disease). In addition, MS patients with chronic progressive (including primary progressive and secondary progressive) forms of the disease present several humoral factors related to its progression [16,21,22,23,24]. In a therapeutic plasmapheresis (plasma exchange), a volume of circulating plasma is extracted to eliminate toxic compounds, and is usually substituted by a 5% albumin solution, or occasionally by fresh frozen plasma (from donors) to replace the plasma volume removed, and thereby maintain the volemia [15,22,25]. In this review, we summarized the current scientific evidence for whether plasmapheresis is effective at reducing toxic circulating factors, and improving clinical symptoms in AD and progressive forms of MS; we also analyzed the adverse effects of this technique.

## 2. Results

Seventy-two papers were retrieved from the studies identified by the search strategy; after eliminating duplicates, 40 required further full-text screening, and nine articles fulfilled the search criteria. We summarized the results of this literature review under four headings: (1) protocol of PP tested; (2) the decline in Aβ (for AD) or auto-immunity mediators (for MS) after plasmapheresis; (3) the beneficial clinical effects observed after PP; (4) the safety and adverse effects of this technique. The general characteristics of the articles are summarized in Table 1, and their details are discussed in the following sections. The design of the studies included in this review was observational and experimental (controlled trials), and some had a follow-up evaluation of the therapeutic effects of PP, which lasted twelve months or more [26,27]. Four clinical studies were randomized, blind, controlled and parallel group clinical trials [28,29,30,31,32]. The population in which PP was studied included 52 AD participants, who were mostly women, aged between 55 and 85 years old, with mild-moderate AD according to the Nacional Institute of Neurological and Communicative Disordes and Stroke-Alzheimer´s Disease and Related Disordes Association (NINCDS-ADRDA) criteria [17], and MS patients between 20 and 61 years old.

### 2.1. Plasmapheresis Protocol

The PP was mostly performed using a commercial continuous flow cell separator with technology based on centrifugation or transmembrane filtration. A peripheral or central double lumen access was used, depending on the patient’s individual characteristics. In each PP session, the total plasma volume of the patient was calculated, taking into account sex, body weight, height, and hematocrit. It required a volume of approximately 35–45 mL/kg, for a volume of 2500–3000 mL for a subject weighing 70 kg; the same volume of 5% serum albumin (60–100 mL/min) was generally administered as a replacement fluid (50 g of albumin per liter of plasma replaced), with a concentration of albumin similar to plasma. A variation of this protocol consisted of 60 mL/kg body weight of plasma exchanged for 3.5% albumin in normal saline containing 6.9 mEq Ca2+/L, 1.2 mEq Mg2+/L, and 4 mEq K+/L [19].

### 2.2. Effect of Plasmapheresis on Amyloid Beta Concentration in AD Patients

Two of the three manuscripts on PP in AD patients came from a single clinical trial [29,30]. While the average levels of Aβ40 and Aβ42 in plasma of AD patients did not show a clear behavior pattern associated with the PP procedure in the seven patients included in the pilot study [26], a clear decreasing pattern was observed over time in the 12-month follow-up study, which was more evident for the Aβ40 concentration [29]. The plasma levels of Aβ40 presented a saw-tooth pattern that ranged between, approximately, 100 and 300 pg/mL. Likewise, plasma Aβ42 levels also behaved with a similar saw-tooth pattern, both in the group treated with PE (ranging between 20 and 60 pg/mL) and in the control group. However, in the group treated with PP, the plasmatic levels of this peptide were statistically lower than in the control group after each treatment period, although during the follow-up period of this study, the levels of Aβ42, and the levels of Aβ40 returned to the control group levels. In the cerebrospinal fluid (CSF), while the average levels of Aβ40 and Aβ42 of the seven patients who underwent PP in the pilot study [26] declined during the PP period, this was followed by a gradual increase during the follow-up period, and returned to baseline levels six months after the start of the study. In contrast, in the follow-up study [29], the levels of both peptides during the treatment period remained stable and showed a slight increase in the case of Aβ42. On the other hand, while the mean values of Aβ42 in CSF between the baseline and the end of the treatment phase showed a tendency to increase in comparison with the average levels in the control group, no significant differences for Aβ40 were observed between the two groups of patients [29].

### 2.3. Effect of Plasmapheresis on Blood Immune Factors in Chronic Progressive MS Patients

In MS patients, PP was mainly tested in individuals with the chronic progressive form of the disease [19,31]. PP reduced the concentration of immunoglobulin IgG, IgA, and IgM immediately, and 7 days after PP. In addition, a decline in serum complement C3 and C4 levels was observed after PP [31]. In the PP clinical study of MS patients, immunosuppressive therapy was added during the PP protocol (mainly low doses of cyclophosphamide and other drugs) in order to minimize the rebound increase in antibodies and other proteins removed by PP. The MS patients’ unresponsiveness to IFN therapy could be attributed to the synthesis of serum inhibitory factors to IFN and to lymphokine [19]. A restoration of responsiveness to IFN therapy in 21 out of 24 patients was demonstrated following PP and immunosuppressive therapy, which was accompanied by a normalization of circulating immune complexes and elevated CD4 lymphocyte counts. The concentration of CD8, human leukocyte antigen—DR isotype (HLA-DR) antigen-bearing cells, NK, serum IFN, and monocyte/macrophage cell populations also increased in the PP responders [19]. However, mixed results have been reported following PP, with no effects on human leukocyte antigen (HLA) typing, T-cell subsets, but enhancement of the suppressor-cell functional activity after PP [30]. A recent study confirmed the utility of PP in reducing the blood concentration of neutralizing antibodies to IFN-beta, and therefore restoring the biological activity of IFN-beta in 4 out of 6 MS patients [20]. Unfortunately, the effect on IFN was transient, and lasted for 1–2 months even during the ongoing PP sessions [20].

### 2.4. Clinical Effects Observed after Plasmapheresis in AD and Chronic Progressive MS Patients

This outcome was analyzed by changes in neuropsychological examination, including cognitive, behavioral, neurological, and functional measures. In AD patients, PP showed a tendency towards cognitive stabilization six months after the end of PE sessions, as assessed by the mini-mental state examination (MMSE) and the Alzheimer’s disease Assessment Scale–Cognitive Subscale (ADAS-Cog) [26]. This beneficial effect was confirmed in a subsequent randomized clinical trial (RCT) [29]. In addition, the RCT patients treated with PP showed a significant improvement in language functions compared to the control group, as assessed by the Boston nomenclature test and semantic verbal fluency; this improvement persisted after PP was discontinued. The control group scored better than the PP-treated group in behavioral (based on the neuropsychiatric inventory, NPI) and functional (Alzheimer’s Disease Cooperative Study - Activities of Daily Living, ADCS-ADL) measures [29]. However, the statistical differences were diminished during the observational phase. A possible explanation for this is that PE has a negative impact on activities of daily living during the intensive treatment phases, but it returns to baseline levels once the treatment is complete. Similarly, patients in the control group had better NPI scores than the group treated with PE, although the treated group showed greater improvement than the control group at the end of the observational phase. This indicates that PP can trigger psychiatric symptoms in AD patients, which are either related to the fact that the patients have to live with a catheter inserted in the chest, experience discomfort caused by metabolic alterations related to PE, or both. Indeed, around 50% of the patients in the treated group developed psychiatric symptoms, especially anxiety. In the case of MS, the RCTs by Khatri et al. [30] and [31] showed an improvement of one or more steps on the Kurtzke DSS scale (the gold standard for MS clinical evaluation) in around 60% of the study sample, whereas the improvement in the control group (sham PP) was around 27% of the study sample. The other patients remained stable, and one worsened in the PP group. Importantly, the consistent and objective neurological improvement did not appear until after 10 weeks of PP therapy, and the PP group continued to improve over time until the twentieth week. In the study by Medenica et al. [19], 21 out 24 patients (87.5%) improved by 2 to 4 steps on the Kurtzke DSS, and stabilization and the duration of these beneficial effects ranged between 2 and 8 years. However, in the study by Hauser et al. [27] fewer than 30% of MS patients clinically improved following PP. The beneficial effects of PP are greater in patients with the cerebral form of MS (compared to cerebellar and spinal clinical presentation of MS) [31], and in those with a shorter total duration of the disease [27,31].

### 2.5. Effects on Brain Alterations Induced by Plasmapheresis

Two studies have evaluated the functional and structural brain changes associated with brain Aβ mobilization and cognitive improvement observed in patients treated with PP [26,28]. Neuroimaging analysis showed a significant increase in brain perfusion in both the frontal and temporal areas six months after treatment [26]. As for brain structural changes, a progressive increase in the volume of the hippocampus was observed, although it did not reach a significant p value. On the contrary, the results of a longitudinal study conducted by Cuberas-Borrós et al. [28] showed a reduced total brain and hippocampus volume, in both patients treated with PP and in controls, as expected in the progression of AD. Likewise, the overall analysis of cerebral perfusion with statistical parametric mapping showed a marked stabilization or an absence of progression of perfusion in the PP-treatment group. In MS patients, no changes in the number and dimension of MS lesions were observed in the CNS by magnetic resonance imaging (MRI) [19] or by computerized tomography of the brain [30]. A reduction was observed in some lesions in some patients, but this effect was attributed to a regression of the surrounding lesion edema rather than a decline in the number of brain plaques [19].

### 2.6. Safety and Adverse Effects

In terms of safety and tolerability, only a single study [29] monitored the adverse effects (AE) that patients experienced during the study period. These authors observed a higher incidence of AE, related to treatment or study procedures or otherwise, in the group that received PP than in the control group (94.7% in plasmapheresis-treated patients versus 70.0% in controls). However, they found no significant differences in the severity of AE (15.8% versus 10.0%). In both groups, the most frequent AE were infections (55.6% versus 28.6%) and psychiatric disorders (50.0% versus 35.7%). The only fatal AE (myocardial infarction) occurred in the group treated with PP, when the patient died two days after undergoing a PP session. However, the researchers believed they were unlikely to be related to the treatment or study procedures. In patients with MS, vascular access was a problem in 18 (out of 26) patients, but was always relieved by a simple technique involving femoral vein catheterization. Eleven of 26 patients [31], or some patients [19], experienced transient hypotension, corrected by rapid infusion of normal saline and 5% albumin solution [19,31]. Two bedridden patients had deep vein thrombosis requiring anticoagulation, but this was probably due to being bedridden rather than PP, per se [31]. One of the six patients switched to centrifugal PP due to an excessive itchy rash (which was resistant to dexamethasone and clemastine treatment). One patient had urticaria, which completely regressed after antihistamine treatment. One patient (out of 6) had appendicitis that was probably unrelated to PP [20].

## 3. Discussion

The knowledge that the main toxic factors for AD pathology are the accumulation of Aβ in the brain, and circulating Aβ peptides crossing into the brain, and contributing to neurological impairment, led to tests of how PP can reduce the Aβ burden in AD patients in a pioneering study by Boada et al. [26]. Considering that 90% of circulating Aβ is linked to albumin, a mobilization of plasma Aβ after PP could induce a mobilization of brain Aβ, and a therapeutic effect in AD patients could consequently be observed [33]. This may indicate that the removal of Aβ proteins, and perhaps other unknown proteins by PP, initiates a more long-lasting process, or processes in the CNS, with beneficial effects on cognition. In fact, these changes in Aβ peptide concentration are associated with cognitive stabilization in a subgroup of AD patients assessed by the MMSE and the ADAS-Cog [26]. In the cognitive subdomains, a significant improvement was observed for some brain functions, e.g., in language and semantic verbal fluency [29]. Importantly, the beneficial effects afforded by PP in language persisted after the PP was discontinued. This mild but significant clinical improvement is accompanied by lower rates of brain hypoperfusion at the frontal and temporal cortex level [26]. Hypoperfusion in AD brains is associated with both structural and functional changes, and is a promising putative biomarker for exploring treatments to slow the progression of the disease when it has become established [34]. An antagonizing effect of PP towards the loss of hippocampal volume also seemed to take place over time when assessed six months after PP, although it did not attain a statistically significant value. The hippocampus, which comprises a number of anatomically interconnected and functionally distinct subfields in the temporal lobes, plays a central role in Alzheimer’s disease and is a crucial mediator of episodic memory [35]. However, the loss of volume in the hippocampus depends on the AD phenotype [36,37,38], and the lack of a significant effect of PP on this neuroimaging parameter could be due to possible different effects on the brain in AD patients with different phenotypes. This aspect clearly warrants future studies. The published evidence for the use of PP in AD is currently limited, and preliminary data from the recently concluded phase IIb/III Alzheimer Management by Albumin Replacement (AMBAR) study are available in abstract form, but has not yet been published in a peer-reviewed journal.

In the case of MS, PP has been tested in patients with the chronic progressive form of the disease and with concomitant immunosuppressive therapy in order to reduce the risk of rebound effects [15,19,27,31]. The course of chronic progressive MS is by definition characterized by a continuous deterioration, and spontaneous remission is rare. Stabilization of improvement would be a significant outcome in patients who are responders to PP. As for the changes induced by PP in MS patients, the markers that have been evaluated belong to the immune system, and hence PP, restores the responsiveness to IFN therapy [19,20], normalizes lymphocyte CD4 counts, CD8, HLADR antigen-bearing cells, NK, serum IFN, and the monocyte/macrophage cell population in PP responders [19]. Unfortunately, the effect on IFN was transient, and lasted for 1 to 2 months even during the ongoing PP sessions [20]. The beneficial effects of PP in MS patients range between 27–87% in clinical studies, and seems to depend on the number of PP sessions and the patient’s characteristics. Analysis of the factors associated with the beneficial effects of PP in MS patients include the cerebral form of MS (compared to cerebellar and spinal clinical presentation of MS) and a total duration of the disease of less than 12 years [30,31], and younger female patients [27]. These results suggest that stratification of MS patients is warranted in future RCT, based on these patients’ features, in order to enhance the advantages of PP. In recent years, PP has also become established as an escalation therapy for steroid-unresponsive relapses of MS, and has thus gained more widespread attention [15]. Clinical improvement rates vary widely between 30% and 80%; these uneven results suggest considerable variability in the efficacy of PP treatment, and show that a large proportion of patients do not respond to PP. This variability in response may be due to disparities in the intensity with which the PP is applied, the number of PP sessions and the speed at which it begins, the type of disease, or the patients’ features in each series. All of these issues should encourage the use of standardized protocols and a detailed analysis of the patients that benefit most from this therapeutic approach.

Possible adverse reactions to PP are mainly related to vascular access, the use of replacement fluids, and the need for anticoagulation [16]. When manifested, the most commonly reported adverse effects observed in patients with immune-mediated neurological disorders are paraesthesias and/or cramps and hypotension [17]. The most frequent AE in both groups were infections and psychiatric disorders. PP can trigger psychiatric symptoms in AD patients, which are either related to the fact that the patients have to live with a catheter inserted in the chest, experience discomfort caused by metabolic alterations related to PP, or both. This aspect should be taken into account in order to monitor behavioral alterations, and eventually, pharmacological treatment during PP protocol. The only mortal AE (myocardial infarction) occurred in the group treated with PP, when the patient died two days after the PP session [29]. In contrast, the MS patients, who were younger than AD patients, presented very few side effects after plasmapheresis.

## 4. Materials and Methods

### Literature Search

We searched the literature in multiple electronic bibliographic databases (Medline, and Cochrane) for all entries until 31 December 2019. The reference lists of all the relevant articles were manually cross-referenced in order to identify additional articles. The primary search terms used were “plasmapheresis”, “apheresis”, and “Alzheimer’s disease” or “chronic progressive multiple sclerosis”. We applied the following inclusion criteria in order to answer the research question: (1) acknowledged as an original article; (2) full text published in either English or Spanish; (3) diagnosis of AD or chronic progressive MS specified by clinical criteria; (4) use of PP with detailed protocol of the technique. The database search results were uploaded into a web-based system, which was used to manage the screening process, and duplicate citations were removed. The members of the review team independently screened the title and abstracts of the articles extracted from the literature search to determine which studies would be included. The full text in electronic format was retrieved for the studies on which the reviewers agreed, based on our inclusion/exclusion criteria. For each of these articles, the two reviewers independently extracted the following data: characteristics of the participants, the AD diagnosis, protocol of PP used, clinical effects of PP, and side effects of PP.

## Figures and Tables

**Table 1:** Main characteristics of clinical studies evaluated.

	Disease/Patients	Subjects (Sex and Age)	Clinical Features	Number of PP or Sham PP Sessions	Effect of Treatment
Chronic progressive multiple sclerosis					
Khatri et al. 1984 and 1985	71 MS patients received PP; 29 MS patients received sham PP.	Age range 23–59 years (mean 37 years, mean percentage 71% female).	Chronic progressive MS. Mean duration of MS: 9.2 years. Mean duration of disease progression 2.1 years.	9–34 sessions (PP frequency: weekly or longer). All received immunosuppressive therapy.	Forty-two patients of 71 (approx. 59%) receiving PP significantly improved on the Kurtzke DSS scale. Eight patients of 29 (approx. 59%) receiving PP significantly improved on the Kurtzke DSS scale.
Hauser et al. 1983	18 MS patients.	Age range 20–52 years (50% female).	Chronic progressive MS. Mean duration of MS: 8.6 years. Mean duration of disease progression 2.9 years.	4–5 sessions (PP frequency: over a two-day period). Control group received ACTH administration.	27.8% improved at 6-month follow-up (22.2% improved at 12-month follow-up). Effect significantly lower compared to the group receiving intensive immunosuppression with high-dose daily i.v. cyclophosphamide plus ACTH (i.v.).
Medenica et al., 1994	24 MS patients	Age range 23–61 years (mean 42 years, 54.2% female).	Chronic progressive MS. Mean duration of MS: 4.2 years. Mean duration of disease progression at least 2 years.	PP was performed for two consecutive days and repeated every 28 days (× 4 times).	Twenty-one patients of 24 (87.5%) significantly improved on the Kurtzke DSS scale. No changes in number and dimension of MS plaques.
Giedraitiene et al., 2015	6 MS patients.	Age range 36–54 years (mean 43.7 years, 66.7% female).	Patients were clinically stable for at least 3 months and were poor responders to IFN-β (autoimmunity). Mean duration of MS: 4.5 years.	4 PP (over 5–8 days). 2.0–2.5 plasma volumes over 5–8 days in each treatment. Donor plasma was used for plasma replacement.	Four of 6 patients (66.7%) regained response to IFN-β therapy, effect was transient: 1–2 months returned to baseline. Two of 6 patients (33.3%) were non-responders (they were the oldest (54 and 49 years old) and had the longest treatment duration (6–7 years).
Alzheimer’s disease					
Boada et al., 2017	18 patients received PP and 19 received sham PP.	Age range 55–85 years (mean age 68 years, 78% female).	Mild-moderate AD according to the probable AD criteria of the NINCDS-ADRDA. Mean duration of symptoms 1.2 ± 0.8 years.	Patients received between 3 and 18 PP or sham PP for 21 weeks administered as follows:	Plasma levels of Aβ42 were significantly lower in the group treated with PP after each treatment period, although these levels tended to return to baseline levels during the observational phase of the study. In addition, patients treated with PP showed significant improvement in language functions, which persisted after the end of the PP protocol.
Cuberas-Borrós et al., 2018	18 patients received PP and 19 received sham PP.	Age range 55–85 years (mean age 68 years, 78% female).	Mild-moderate AD according to the probable AD criteria of the NINCDS-ADRDA. Mean duration of symptoms 1.2 ± 0.8 years.	Patients received between 3 and 18 PP with human albumin 5% or sham PP for 21 weeks administered as follows:	As expected for the evolution of the disease, patients receiving PP showed a decrease in total brain volume and the hippocampus. Furthermore, compared to controls, they had a lower perfusion loss in the frontal, temporal and parietal areas at 6 months after the PP.
Boada et al., 2007 and 2009	7 AD patients.	Age range 55–85 years.	Mild-moderate AD according to the probable AD criteria of the NINCDS-ADRDA.	Patients received between 3 and 5 PP for three weeks, with a frequency of 2 PP sessions per week.	The Aβ Plasma levels showed a clear saw-toothed pattern, more evident for Aβ40, during the treatment period. A tendency towards cognitive stabilization 6 months after the PP was over was also observed. Neuroimaging results showed a significant perfusion increase in both the frontal and temporal areas at six months after treatment
